# Application of machine learning techniques to profile smoking behavior of adolescent girls in Ghana

**DOI:** 10.12688/gatesopenres.14991.1

**Published:** 2024-01-03

**Authors:** Sara V. Flanagan, Ariadna Vargas, Jana Smith

**Affiliations:** 1 ideas42, New York, New York, 10004, USA

**Keywords:** machine learning, synthetic data, smoking, tobacco, adolescent girl, algorithm, behavioral science

## Abstract

**Background:**

Tobacco use trends among adolescents in low- and middle-income countries, and in particular narrowing gender gaps, highlight the need for interventions to prevent and/or reduce tobacco use among adolescent girls. We evaluated a social marketing program in Ghana discouraging tobacco use among adolescent girls and additionally investigated the pathways influencing smoking behaviors to identify programmatic opportunities for impact. Leveraging the data collected through the stepped wedge cluster randomized trial and panel survey of 9000 girls aged 13–19 , we sought to apply machine learning (ML) techniques to identify the most important variables for predicting initiation of smoking.

**Methods:**

To identify predictors of smoking initiation we sought to develop a model which could accurately differentiate smokers from non-smokers and evaluated various ML approaches for training classifier algorithms to achieve this. We selected a Synthetic Minority Over-sampling Technique (SMOTE) because it optimized the recall and precision of the model. We then utilized the technique of feature importance for greater insight into how the model arrived at its decisions and to rank the most important variables for predicting smokers. To explore different dimensions of smoking behavior, including initiation and continuation, we trained our model by using several combinations of target outcomes and input variables from the panel survey.

**Results:**

The resulting features of smokers highlight the importance of girls’ independence and connectivity, social environment, and peer influence on likelihood of smoking, and in particular subsequent initiation. These results were largely consistent with our formative research findings based on qualitative interviews informed by behavioral science.

**Conclusions:**

This novel application of ML techniques demonstrates how data science approaches can generate new programmatic insights from rigorous evaluation data, especially when data collection is informed by behavioral theory. Such insights about the relative importance of different features can be valuable input for program planning and outreach.

## Introduction

Machine learning (ML) is a discipline at the intersection of data science and artificial intelligence with a focus on building algorithms to make predictions without requiring explicit programming to do so ^1^. The approach involves building a model using sample training data. These methods are increasingly being applied to support evidence-based decision making across a wide range of fields, including global health. Here we report our experience applying ML to generate programmatic insight from data collected for a rigorous impact evaluation.

As external evaluators of a social marketing program in Ghana focused on discouraging tobacco use among adolescent girls, we conducted a stepped wedge cluster randomized trial and panel survey of 9000 girls aged 13–19 in select neighborhoods of Accra and Kumasi over twelve months of 2021–2022. A secondary objective was to provide additional behavioral insights on the pathways influencing smoking behavior among teenage girls in Ghana and to identify programmatic opportunities for impact. Although a formative research phase with qualitative interviews informed by the behavioral science literature was conducted to support this objective, we were interested to explore how recent advances in ML techniques might be applied to our panel survey data to generate additional insights related to the predictors of smoking initiation and to compare and contrast findings from these two approaches.

Although our study had a relatively large sample, smoking behavior was quite rare. Only 1.6% of girls reported having ever tried smoking (defined as cigarette or shisha use) at baseline and 0.1% having smoked in the past 30 days. We wanted to think more creatively about ways to profile potential smokers in this population beyond basic demographic slices of the sample. Our objective was to first build an algorithm that would predict which girls are more likely to be smokers and then identify which factors are most important to make those predictions. In this research note we describe how we explored several ML methods to build a classifier model and then applied a ML explainability method to profile the most important predictors of smokers using different smoking definitions, as well as different data subsamples.

## Methods

### Data source

Data collection for the step wedge evaluation included a survey of all 9000 girls over four rounds—Baseline (before any implementation), Midline 1 (after the first period of implementation), Midline 2 (after the second), and Endline (after all areas had been activated). Participants aged 13–19 were recruited through multi-stage sampling in which neighborhood clusters in Accra and Kumasi were first randomly selected for the study, and then households within each cluster were systematically approached through a community mapping process until the target number of adolescent girls willing to participate in the study were identified. To be considered eligible for the study, girls had to have access to a phone and had to intend to remain at their residence over the subsequent year, to enable enumerators to reach them at future rounds of the panel survey. The questionnaire was informed by the formative research and a smoking-oriented theory of change and comprised about 100 questions covering: background and demographic information; social context, confidence, and self-efficacy; sources of influence on girls’ decisions and actions; tobacco perceptions and norms; tobacco use, opportunity, and refusal; and program exposure and perceptions. The survey was reviewed for face validity with the program team and portions were piloted with 396 adolescent girls during the formative research phase. Most responses were binary or Likert scales of 1 to 4 that were recoded to binary (agree vs. disagree, likely vs. not likely) for this exploratory exercise. Several indices of multiple survey items that were confirmed to have high internal consistency served as indicators of intermediate outcomes in the impact evaluation; however, for this application of ML techniques survey items were maintained as individual variables. Ethical review and approval for the evaluation study were provided by Innovations for Poverty Action IRB (protocol #15798) and the Ghana Health Service Ethics Review Committee (GHSERC: 003/11/20). Written informed consent to participate in the study was obtained from participants aged 18 years or older. For younger participants, written parental consent was first obtained followed by written assent of the adolescent.

### Building the classification model

**Figure 1.**
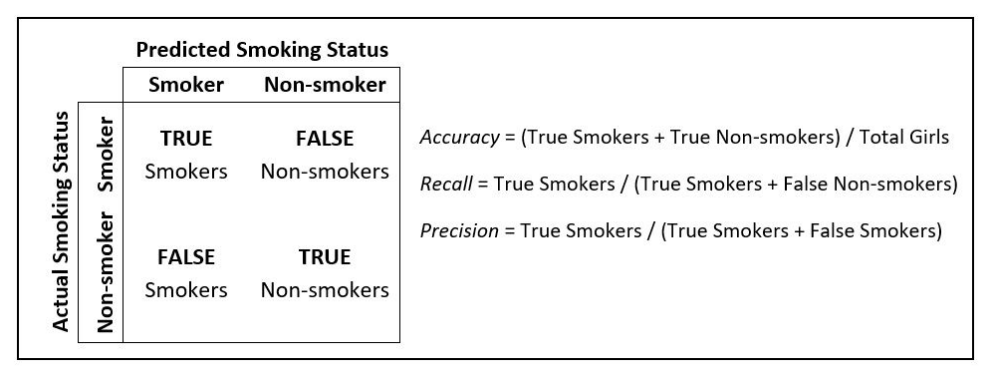
Actual vs. predicted smoking status.

Initially, we trained a basic Random Forest classifier using the baseline data to identify girls who have ever smoked, which performed well in terms of accuracy ( Table 1). Accuracy is defined as the proportion of all girls who are correctly classified as either smokers or non-smokers ( Figure 1).

**Table 1.** Performance of classification algorithm using machine learning techniques.

Model	Accuracy	Precision	Recall
Without oversampling	0.9594	0.2500	0.0448
RandomOverSampler	0.9586	0.6494	0.3117
ADASYN	0.9669	0.5445	0.2675
SMOTE	0.9901	0.7900	0.7299
Class weighting	0.9174	0.3576	0.2595
Hellinger Distance	0.9131	0.7413	0.6603

To optimize our baseline model, we chose to focus on increasing the recall metric, rather than just accuracy. Recall is a measure of the model's ability to correctly identify actual smokers; a high recall value indicates that the model is able to identify most girls who smoke, while a low recall value suggests that the model is missing a significant number of smokers. Accuracy, which is affected by the low prevalence of smoking in our data, can bias the model towards classifying most individuals as non-smokers. Therefore, we were more concerned with correctly identifying those who did smoke to ensure effective programmatic targeting towards likely smokers, even if it meant that some non-smokers were mistakenly identified as smokers.

Additionally, we focused on improving the model’s precision. If the precision of the model is low, it means that the model is classifying many individuals as smokers even though they are not, which could result in potentially unnecessary or less well-targeted interventions. Therefore, improving precision helps to reduce targeting of non-smokers and ensures that the resources allocated to smoking interventions are still used effectively and efficiently.

With the objective of optimizing the recall and precision, we evaluated the following ML approaches using the “imbalance-library” Python package ^2^ (which is built upon the “scikit-learn” ML library) to determine which method produced the optimal outcome:

- *RandomOverSampler*: This technique works by randomly oversampling the minority class (non-smokers) in the dataset, i.e., it generates additional random samples of the minority class to balance the class distribution.
- *ADASYN (Adaptive Synthetic Sampling)* ^3^: ADASYN works by generating synthetic samples of the minority class based on the density of the minority class in the feature space and the distance between minority samples. The goal is to increase the number of samples of the minority class in the training data while maintaining a balanced representation of the classes in the feature space.
- *SMOTE (Synthetic Minority Over-sampling Technique)* ^4^: Like ADASYN, SMOTE works by generating synthetic samples of the minority class, but instead of generating samples based on the density and distance of the minority class, it generates samples based on the k-nearest neighbors of the minority samples.
- *Class weighting*: This method assigns higher weights to the minority class, encouraging the algorithm to pay more attention to the minority class and make better predictions.
- *Hellinger Distance as a Tree Split Criterion* ^5^: Hellinger distance is a measure of the difference between two probability distributions. In decision tree algorithms, Hellinger distance can be used as a criterion for splitting nodes in the tree, to determine the most informative split that maximizes the difference between the classes in the feature space. Using Hellinger distance as a split criterion can improve the accuracy of decision tree algorithms in imbalanced classification tasks by giving more attention to the minority class.

We ultimately selected the SMOTE technique of generating synthetic data for training the classification algorithm because it optimized its performance in terms of recall and precision ( Table 1).

### Generating smoker profiles

After successfully building a high-performing classification model, our goal was to build a profile of girls who smoke. However, ML models are often referred to as "black boxes" because they can be difficult to interpret and comprehend. This means that the model might make accurate predictions, but it's not clear how it arrived at those predictions or why a particular prediction was made.

To gain more transparency, we utilized the technique of feature importance. This method explains predictions made by the model by identifying which features or input data had the most significant impact on those predictions. Essentially, it helps us understand which characteristics have the most influence in predicting a girl to become a smoker. By calculating feature importance we aimed to generate greater insight into how the model arrived at its decisions and to rank the most important variables. We determined whether an important feature is a risk for or protective against smoking based on the direction of that variable’s association with the smoking outcome in our data.

To determine the profiles of girls most likely to smoke, we trained our model by using the following combinations of target outcomes and input variables from our survey data:

Model #1: Predicted outcome: reported ever tried smoking at baseline.
  Training inputs: baseline survey responses
Model #2: Predicted outcome: reported having smoked during the previous month at endline.
  Training inputs: endline survey responses
Model #3: Predicted outcome: among non-smokers at baseline, reported ever tried smoking at endline.
  Training inputs: baseline survey responses

As a secondary analysis we looked at these same combinations within sub-groups of respondents, including younger vs. older girls and by region, to support interpretation of findings and identify heterogeneity in predictors. We also analyzed additional combinations of data inputs and predicted outcomes for robustness.

## Results

Model #1 ( Table 2) differentiates between girls based on smoking history prior to the study. The results highlight that girls who had ever tried smoking report different social settings and experiences at present than non-smokers. They are more likely to have regular phone access, recently attended a party, and/or been recently offered alcohol, which suggest more independence and social activity. They are also more likely to think their close friends have tried shisha, that their friends would approve of them smoking shisha, and to say that they would not likely refuse an offer of shisha from friends. Several indicators included in the evaluated program’s theory of change, particularly those related to girls’ confidence in expressing preferences to friends, were not important to classifying likely smokers.

**Table 2.** Model 1, reported ever smoking at baseline.

Variable Input	Importance
Went to a party last 30 days (+)	0.128
Thinks most friends smoke shisha (+)	0.084
Has regular access to phone (+)	0.069
Would refuse shisha from friends (-)	0.053
Was offered alcohol last 30 days (+)	0.043
Friends would approve smoking shisha (+)	0.040
Thinks most other girls smoke shisha (+)	0.040
Lives in Accra (+)	0.034
Age (+)	0.033
Friends would shun if smoked shisha (-)	0.032
Friends would shun if smoked cigarette (-)	0.032
Went to a bar last 30 days (+)	0.028
Would refuse shisha from boyfriend (-)	0.024
Lives with both parents (-)	0.022
Believes smoking shisha is harmful (-)	0.022
Friends would approve smoking (+)	0.021
In school (-)	0.019
Parents influence their decisions (-)	0.017
Has close circle of friends (-)	0.017
Believes not smoking shisha is important (-)	0.016
Thinks people who say no are admired (+)	0.014
Friends would shun if said no (+)	0.013
Boyfriend would shun if said no (+)	0.013
Friends influence their decisions (+)	0.012
Others think smoking cigarettes is guy-guy (+)	0.012
Most activities involve smoking shisha (+)	0.012
Others think smoking shisha is guy-guy (+)	0.011
Girls like other confident girls (-)	0.011
Boys like confident girls (-)	0.010
Most activities involve smoking cigarettes (+)	0.010
Believes smoking cigarettes is harmful (-)	0.009
Boys like girls who smoke shisha (+)	0.009
Would refuse cigarette from friends (-)	0.007
Feels comfortable expressing likes/dislikes (-)	0.007
Stands firm with friends (-)	0.006
Would refuse cigarette from boyfriend (-)	0.005
Thinks most other girls smoke cigarettes (+)	0.005
Boys like girls who smoke cigarettes (+)	0.005
Can tell friends if uncomfortable (+)	0.005
Believes not smoking cig is important (-)	0.003
Think most friends smoke cigarettes (+)	0.003
Other girls influence their decisions (+)	0.001

Note: +/- indicate whether that variable is associated with increased or decreased risk of smoking

Given that smoking in this population is rare and infrequent, Model #2 ( Table 3) identifies which factors are most important to differentiate recent smokers from recent non-smokers. Endline responses were used given very few recent smokers at baseline. Recent smokers are more likely to be older and live in the capital Accra and report lower parental influence than non-smokers, another sign of greater independence. They are more likely to report that smoking is common among their peers and that they would accept an offer to smoke from friends, and less likely to perceive shisha as harmful. The belief that people who say no are admired by others is one of the top factors predictive of being a recent smoker; this may suggest positive views of smoking as demonstrating independence or rebelliousness against norms.

**Table 3.** Model 2, recent smoker at endline (abbreviated).

Variable Input	Importance
Age (+)	0.120
Thinks most other girls smoke cigarettes (+)	0.108
Lives in Accra (+)	0.106
Would refuse cigarette from friends (-)	0.090
People who say no are admired (+)	0.079
Parents influence decisions (-)	0.060
Thinks smoking shisha is harmful (-)	0.045
Went to a party last 30 days (+)	0.035
Friends would shun if smoked shisha (-)	0.031
Has close circle of friends (-)	0.030
Think most friends smoke shisha (+)	0.029
Lives with both parents (-)	0.027
Most activities involve smoking shisha (+)	0.022
Most activities involve smoking cigarettes (+)	0.018

Note: +/- indicate whether that variable is associated with increased or decreased risk of smoking

From Model #3 ( Table 4) we profiled the small number of girls who were non-smokers at baseline but reported they had tried smoking by endline, in order to identify which factors are most important to set them apart from girls who remained non-smokers during the study period. We see that age and phone access were most important to subsequent smoking initiation, which suggests that these girls may already have had more independence and connectedness than other non-smoking girls. The girls that started smoking within the study period were also more likely to perceive that most social activities for girls their age involve shisha and that most girls their age have tried shisha, which suggests that they are also experiencing or perceiving a different social environment at baseline than other non-smokers. And these girls are more likely to report their friends have influence on their decisions, and less likely to report parent influence than other non-smokers, which suggests they might be more susceptible to social opportunity and persuasion.

**Table 4.** Model 3, non-smoker at baseline, smoked by endline (abbreviated).

Variable Input	Importance
Age (+)	0.129
Has regular access to phone (+)	0.079
Friends influence decisions (+)	0.053
In school (-)	0.050
Lives with both parents (-)	0.041
Most activities involve smoking shisha (+)	0.037
Parents influence decisions (-)	0.035
Think other girls smoke shisha (+)	0.033
People who say no are admired (+)	0.031
Has close circle of friends (-)	0.030
From Accra (+)	0.028
Most activities involve smoking cigarettes (+)	0.028
Friends would shun if said no (+)	0.027

Note: +/- indicate whether that variable is associated with increased or decreased risk of smoking

## Conclusion/discussion

We report here a novel application of ML techniques to generate insights around a behavior that is still relatively rare among urban teen girls in Ghana. Creating synthetic data through the SMOTE technique created a more balanced training data set for the classifier, and by opening up the black box of the algorithm we learned which features are most important towards predicting girls likely to be smokers.

While very few girls in Ghana have tried smoking, even fewer report having smoked in the previous 30 days. Models 1 and 2 suggest both groups are more likely to be older and have the freedom to attend social events where they may be exposed to smoking opportunities or behaviors that shape their perceptions of norms among peers. However, a perceived higher prevalence of cigarette smoking and willingness to accept cigarettes from friends are distinctively strong predictors of recent smokers. This may reflect that shisha is more commonly experimented with among girls this age and is more likely to be smoked on an infrequent basis (less than monthly). For that reason, most strong predictors of ever having smoked reflect beliefs about shisha, whereas recent smokers who smoke cigarettes, while still the minority, may have particularly strong and distinctive views on cigarette smoking. Other distinctively strong predictors of recent smoking include reporting low influence of parents and expressing admiration for people who are willing to go against trends. Parents in Ghana are generally highly protective of their teenage girls and there is a strong social norm against smoking ^6^; these findings may reflect the absence of such parental figures in smokers’ lives, or their willful rebellion against them.

Model 3 supports that changes in independence and the social environments shaping girls’ opportunities to smoke do in fact precede initiation and experimentation. Cross-sectional surveys can support associations between smoking behavior and social environment; the Model 3 classifier leverages survey data from two timepoints to highlight the pre-existing differences among non-smoking girls that are most predictive of subsequent smoking initiation. Among younger girls, this may reflect different home environments, where girls have more mobility and less parental oversight, putting some girls at more risk of smoking opportunities. These differences may also explain why older age is such a strong predictor of smoking, as adolescent girls are often given more freedom to attend social events and activities as they age. Furthermore, many girls in urban Ghana enroll in a boarding school for their Senior High School (SHS) education where they may gain exposure to girls from less sheltered backgrounds who may influence subsequent opportunities for smoking ^6^.

Such insights about the relative importance of different features to a target behavior can be valuable input for program planning and outreach. This is especially true in the case of programs aimed at smoking prevention, given that smoking in teens is addictive and the literature suggests that a majority who start at this age will continue to do so in adulthood ^6^. Better understanding of the risk factors for recent and future smoking behavior, and in particular the role of the social environment, can suggest programmatic opportunities for targeting adolescents most at risk or exploring more promising programmatic directions, such as reshaping the environments they are exposed to.

Although the smoker profiles generated through this approach are limited by the inputs we provide the machine, i.e., our survey data, they were largely consistent with our formative qualitative research findings around influences on girls’ behavior ^5^. Data collection tools in both cases were informed by insights from the tobacco literature and behavioral science; we believe grounding survey data collection in strong behavioral science theory strengthened the utility of the ML outputs and resulted in better alignment of findings with more in-depth qualitative research techniques. This novel application of ML techniques demonstrates the potential synergism between data science and behavioral science to generate insights about predictors of behavior and highlights the importance of basing quantitative data collection in behavioral theory, especially if opportunities for rich qualitative investigation are limited in other settings.

## Data Availability

The data that support the findings of this study are not publicly available due to privacy and ethical restrictions but are available from the corresponding author on reasonable request. A reasonable request would be from a legitimate party with a specific research objective, would not violate the privacy or ethical protections of participants, and would not require additional reformatting or repackaging of the data by the authors. The baseline survey questions are available here: https://doi.org/10.6084/m9.figshare.24581616 ^7^ This project contains the following extended data: Baseline Survey_Final.pdf Data are available under the terms of the Creative Commons Attribution 4.0 International license (CC-BY 4.0).

## Software availability

The RandomForestClassifier that was used for classification tasks belongs to the open-source Python scikit-learn library ^8^ available at https://github.com/scikit-learn/scikit-learn, with documentation here: https://scikit-learn.org/stable/modules/generated/sklearn.ensemble.RandomForestClassifier.html. The feature importances are provided by the "feature_importances" attribute of the scikit-learn's RandomForestClassifier, which offers a way to assess the importance of each feature in making accurate predictions with the random forest model.

All the oversampling methods were performed using open-source Python imbalanced-learn library ^2^ hosted at https://github.com/scikit-learn-contrib/imbalanced-learn with documentation here: https://imbalanced-learn.org/stable/references/generated/imblearn.over_sampling.RandomOverSampler.html.
