# Supplementary material for: Application of machine learning techniques to profile smoking behavior of adolescent girls in Ghana
Source: Gates Open Res. 2024 Nov 20;8:2. Originally published 2024 Jan 3. [Version 2] doi: 10.12688/gatesopenres.14991.2 (PMC12381352; doi:10.12688/gatesopenres.14991.2)
Supplement: Peer review history

# Reviewer report: Reviewer response for version 2

**DOI:** 10.21956/gatesopenres.17698.r39728

**Published:** 2025-08-26

**Authors:** Runqiu Wang

**Affiliations:** 1 University of Nebraska Medical Center, Omaha, Nebraska, USA

The paper "Application of machine learning techniques to profile smoking behavior of adolescent girls in Ghana" applies machine learning (ML) methods, mostly random forest classifier, to identify predictors of smoking initiation among adolescent girls using data from a stepped wedge cluster randomized trial and panel survey involving 9,000 girls aged 13-19 years. The study leverages the Synthetic Minority Over-sampling Technique (SMOTE) to optimize model precision and recall and employs feature importance techniques to enhance interpretability.

 Below are my comments:

 1.Introduction: Strengthen the introduction by briefly discussing current tobacco use prevalence among adolescents in Ghana and the gender gap trend. Cite recent literature to contextualize the relevance and urgency of this issue, for example:

 a. (Ref 1)

 b. (Ref 2)

 2. Methods:

 (1).Suggest including results from a conventional logistic regression model as a baseline comparison. This would provide a useful benchmark and highlight the specific advantages or limitations of the Random Forest classifier in this context.

 (2).While the paper presents itself as applying machine learning broadly, only the Random Forest classifier is used. The authors may consider exploring additional ML models: such as gradient boosting machines (e.g., XGBoost), support vector machines to assess model robustness and validate the consistency of key predictors. This would strengthen the claim that machine learning approaches (not just Random Forests) offer valuable insights in this context.

 (3) The manuscript does not describe the validation strategy used for model training and evaluation. It is unclear whether cross-validation, train/test split, or another method was used to compute the reported metrics (accuracy, precision, recall). Clarifying the validation framework and specifying how these metrics were calculated would strengthen the credibility and reproducibility of the results.

 (4) SMOTE should only be applied to the training set to avoid data leakage. The authors should clarify whether SMOTE was applied correctly after splitting the data and not on the entire dataset. Improper use could compromise the validity of performance metrics and model generalizability.

 3. Results:

 The reported feature directions (positive or negative associations) are not derived from the Random Forest model itself, as standard feature importance metrics do not provide directionality. The authors should clarify how these directions were determined, e.g., through separate bivariate analyses, and discuss the limitations of inferring directionality outside the trained model context. Alternatively, the authors may consider using SHAP (SHapley Additive exPlanations). SHAP provides both the direction and magnitude of each feature’s contribution to a prediction, offering a unified and theoretically grounded method for interpreting machine learning models.

 4. Conclusion/Discussion

 I have no comments here, authors did a good job for this part.

Is the work clearly and accurately presented and does it cite the current literature?

Partly

If applicable, is the statistical analysis and its interpretation appropriate?

Yes

Are all the source data underlying the results available to ensure full reproducibility?

Partly

Is the study design appropriate and is the work technically sound?

Yes

Are the conclusions drawn adequately supported by the results?

Yes

Are sufficient details of methods and analysis provided to allow replication by others?

Partly

Reviewer Expertise:

Biostatistics, Machine Learning and Deep Learning, multiple testing in high-dimensional data, infectious disease

I confirm that I have read this submission and believe that I have an appropriate level of expertise to confirm that it is of an acceptable scientific standard, however I have significant reservations, as outlined above.

## References

[1] : Social norms and social opportunities: a qualitative study of influences on tobacco use among urban adolescent girls in Ghana. *BMC Public Health* .2024;24(1) : 10.1186/s12889-024-20413-z 10.1186/s12889-024-20413-z 39468503 PMC11514744

[2] : Profile and predictors of adolescent tobacco use in Ghana: evidence from the 2017 Global Youth Tobacco Survey (GYTS). *J Prev Med Hyg.* . 10.15167/2421-4248/jpmh2021.62.3.2035 10.15167/2421-4248/jpmh2021.62.3.2035 PMC863912434909494

# Reviewer report: Reviewer response for version 2

**DOI:** 10.21956/gatesopenres.17698.r38488

**Published:** 2024-12-11

**Authors:** Ruoyan Sun

**Affiliations:** 1 The University of Alabama at Birmingham, Birmingham, Alabama, USA

The authors have addressed most of my previous comments. I have two minor questions. 

 1. In the Introduction, new references are added as footnotes (Footnote I to VI) but not cited. Is this because of limited references allowed (up to 8)? If there is space for more than 8 references, then the authors should cite these new references properly. 

 2. Tysa & Pederson (Footnote vi) was published in 1998 and smoking behaviors have changed substantially since then in many countries. Is there any more recent references that can be used?

Is the work clearly and accurately presented and does it cite the current literature?

Partly

If applicable, is the statistical analysis and its interpretation appropriate?

Yes

Are all the source data underlying the results available to ensure full reproducibility?

Yes

Is the study design appropriate and is the work technically sound?

Yes

Are the conclusions drawn adequately supported by the results?

Yes

Are sufficient details of methods and analysis provided to allow replication by others?

Yes

Reviewer Expertise:

Tobacco control; policy evaluation; health economics; modeling and simulation.

I confirm that I have read this submission and believe that I have an appropriate level of expertise to confirm that it is of an acceptable scientific standard.

# Reviewer report: Reviewer response for version 1

**DOI:** 10.21956/gatesopenres.16324.r36854

**Published:** 2024-07-17

**Authors:** Jennifer M. Murray

**Affiliations:** 1 Queens University Belfast, Belfast, England, UK

This Research Note applies machine learning (ML) techniques to identify the most important variables for predicting initiation of smoking amongst 9000 adolescent girls (aged 13-19 years) participating in a social marketing program in Ghana. The data was collected through a stepped wedge cluster randomized trial with panel survey over four time-points during 12 months of 2021-2022. The results show the importance of adolescent girls' independence and connectivity, social environment, and peer influence for predicting previous smoking behavior, and subsequent smoking initiation. The study design and methods are appropriate for answering the research questions, and the work is technically sound. The statistical analysis and its interpretation are appropriate, and sufficient details of the methods and analysis have been provided to allow replication. The conclusions drawn are adequately supported by the results. The source data underlying the results are not publicly available due to privacy and ethical restrictions. However, the authors have said that they will provide the data on reasonable request. The authors have also provided a link to the survey questions in the "Extended data" section. Overall, the work is clearly and accurately presented. However, there is little reference to the current literature on adolescent smoking initiation or the behavioral theory underlying the study and outcome measures. I believe that the work is OK to be indexed in its current form, but I have chosen the "approved with reservations" approval status because I have several minor comments that I think could improve the published product.

 Key strengths: Large sample size, methods are appropriate for answering the research questions, comprehensive dataset with outcome variables and predictors informed by behavioral theory and qualitative research findings.

 Key weaknesses: Little discussion of the current literature on adolescent smoking or the relevant research on behavioral theory.

 Comments:

  

1. There is little discussion on the current literature on adolescent smoking in the introduction section of the main text. For example, although the background section of the abstract mentions tobacco trends in low- and middle-income countries, and narrowing gender gaps, this is not expanded upon in the main text and there is no mention of the adolescent smoking prevalence (for girls in Ghana). I appreciate that the article is a Research Note and therefore has a limited word count, but I would still expect to see some reference to the issue of adolescent smoking rates in the introduction section.
2. Similarly, throughout the paper the authors highlight that their survey items (and the ML inputs) were informed by the formative/qualitative research findings, and the behavior change theory underlying the evaluated program. However, the qualitative findings or the behavioral theory have not been summarized anywhere. I think it would be interesting to see a brief overview of the social marketing program, and how it relates to the study's outcome measures, in the methods section.
3. At the end of the methods section, you state that you conducted some secondary analyses using the same data inputs and predicted outcomes within subgroups of the respondents (including younger versus older girls, and different regions), and also used different combinations of data inputs and predicted outcomes. However, you were not very specific about these analyses, and the results are not reported. I suggest you should add specific details about these analyses (e.g., what age groups and regions were compared, what alternative combinations of data inputs and predicted outcomes were used) and summarize the findings in the results section. You could also upload the results tables for the secondary analyses to a repository and provide the link in the "Extended data" section. I do not think this should be left as it is because it is vague, and the results have not been discussed anywhere in the article. It would be interesting to see the results if you had used the "intentions for smoking cigarettes or shisha over the next 30 days" outcome at the end of the survey.
4. Similarly, in the methods section you state that you recoded some of your data's Likert scales to binary. This would cause some loss of information. Have you considered presenting alternative (sensitivity) analyses to determine the impact of using the original uncategorized outcome variables?
5. Although you have provided a link to the full survey in the "Extended data" section, I think you could provide some more information on the outcome measures in the methods section (e.g., insert the relevant references if they have been adapted from previous research studies, and describe whether they have been validated for use with your research population).
6. The study's strengths and limitations have not been discussed in the discussion section.
7. On page 4, where you provide an overview of the ML techniques, you state that the "RandomOverSampler" technique works by "randomly oversampling the minority class (non-smokers)". Are smokers not the minority class?
8. Where you describe the SMOTE technique (the optimal technique whose results you ultimately report on), you say that it works by generating samples based on the k-nearest neighbors of the minority samples. What was "k" in your models?
9. In your models, "lives in Accra" is frequently an important predictor. Can you comment on this result (e.g., the differences between Accra and the other communities in your sample, and why adolescents living in Accra should have increased risk of smoking)?

Is the work clearly and accurately presented and does it cite the current literature?

Partly

If applicable, is the statistical analysis and its interpretation appropriate?

Yes

Are all the source data underlying the results available to ensure full reproducibility?

Partly

Is the study design appropriate and is the work technically sound?

Yes

Are the conclusions drawn adequately supported by the results?

Yes

Are sufficient details of methods and analysis provided to allow replication by others?

Yes

Reviewer Expertise:

Public health, health behavior change, physical activity, adolescent smoking, peer influence, mediation analyses, SIENA modelling

I confirm that I have read this submission and believe that I have an appropriate level of expertise to confirm that it is of an acceptable scientific standard, however I have significant reservations, as outlined above.

# Response

**Published:** 2024-11-19

**Authors:** Sara Flanagan

**Affiliations:** ideas42, USA

1. There is little discussion on the current literature on adolescent smoking in the introduction section of the main text. For example, although the background section of the abstract mentions tobacco trends in low- and middle-income countries, and narrowing gender gaps, this is not expanded upon in the main text and there is no mention of the adolescent smoking prevalence (for girls in Ghana). I appreciate that the article is a Research Note and therefore has a limited word count, but I would still expect to see some reference to the issue of adolescent smoking rates in the introduction section.
  - **Response**: This background info has been added to the Introduction section of the main text along with citations.
2. Similarly, throughout the paper the authors highlight that their survey items (and the ML inputs) were informed by the formative/qualitative research findings, and the behavior change theory underlying the evaluated program. However, the qualitative findings or the behavioral theory have not been summarized anywhere. I think it would be interesting to see a brief overview of the social marketing program, and how it relates to the study's outcome measures, in the methods section.
  - **Response**: The qualitative study has just recently been published and the citation has been updated. That paper includes more explanation of the context for the study and an extensive discussion of the findings relative to the literature.
3. At the end of the methods section, you state that you conducted some secondary analyses using the same data inputs and predicted outcomes within subgroups of the respondents (including younger versus older girls, and different regions), and also used different combinations of data inputs and predicted outcomes. However, you were not very specific about these analyses, and the results are not reported. I suggest you should add specific details about these analyses (e.g., what age groups and regions were compared, what alternative combinations of data inputs and predicted outcomes were used) and summarize the findings in the results section. You could also upload the results tables for the secondary analyses to a repository and provide the link in the "Extended data" section. I do not think this should be left as it is because it is vague, and the results have not been discussed anywhere in the article. It would be interesting to see the results if you had used the "intentions for smoking cigarettes or shisha over the next 30 days" outcome at the end of the survey.
  - **Response: **We have updated the methods to specify the age groups (13-15 vs. 16-19) and regions (Accra vs. Kumasi), and summarized the age subgroup results for model 3 at the end of the results section. We also edited the Methods so that it does not seem like we explored additional outcomes (we did not explore intention to smoke), rather we looked at the same outcomes (ever smoking, recent smoking) at different time points.
4. Similarly, in the methods section you state that you recoded some of your data's Likert scales to binary. This would cause some loss of information. Have you considered presenting alternative (sensitivity) analyses to determine the impact of using the original uncategorized outcome variables?
  - **Response: **SMOTE uses Euclidean distance to figure out how close minority samples are to each other, but can struggle with high-dimensional or ordinal data, like raw Likert scales, because of a problem called “distance distortion.” In high-dimensional spaces, data points are more spread out, which makes their distances more similar and makes it hard to find true nearest neighbors. This distortion could make a sensitivity analysis confusing, since it will be difficult to know if any differences are from real predictive value or just from high-dimensional effects. Moreover, transforming the Likert scale variables was also to make interpretation of the feature importance technique easier, so using the original scales again may not add much value, given potential distortion, and could reintroduce complexity to the interpretation.
5. Although you have provided a link to the full survey in the "Extended data" section, I think you could provide some more information on the outcome measures in the methods section (e.g., insert the relevant references if they have been adapted from previous research studies, and describe whether they have been validated for use with your research population).
  - **Response: **We added a comment that the tobacco use questions were adapted from the Global Youth Tobacco Survey, and cited the formative research, which is the context in which the questionnaire was tested in this population.
6. The study's strengths and limitations have not been discussed in the discussion section.
  - **Response: **The final paragraph of the discussion mentions the limitations of data inputs and the strengths of grounding data collection in behavioral science. We have expanded there to mention the large sample size as a strength and referenced a longer discussion of limitations related to collecting data from this study population in our formative research paper, to save on space here.
7. On page 4, where you provide an overview of the ML techniques, you state that the "RandomOverSampler" technique works by "randomly oversampling the minority class (non-smokers)". Are smokers not the minority class?
  - **Response: **This was a typo and has been corrected, we thank the reviewer for catching it.
8. Where you describe the SMOTE technique (the optimal technique whose results you ultimately report on), you say that it works by generating samples based on the k-nearest neighbors of the minority samples. What was "k" in your models?
  - **Response: **We used 5 k-neighbors and added this detail to the description of the technique.
9. In your models, "lives in Accra" is frequently an important predictor. Can you comment on this result (e.g., the differences between Accra and the other communities in your sample, and why adolescents living in Accra should have increased risk of smoking)?
  - **Response: **We have added a comment to the results discussion that Accra is the more cosmopolitan and diverse of the two study cities.

# Reviewer report: Reviewer response for version 1

**DOI:** 10.21956/gatesopenres.16324.r36278

**Published:** 2024-05-16

**Authors:** Ruoyan Sun

**Affiliations:** 1 The University of Alabama at Birmingham, Birmingham, Alabama, USA

Applying novel machine learning techniques to a panel survey of 9000 girls aged 13-19 in Ghana, this study identified important variables that predict smoking initiation among adolescent girls. Strengths of the study include clearly written descriptions of various machine learning approaches and model selection based on performance measures (accuracy vs prevision vs recall). However, there are a few problems.

 1. There is a lack of information or justification of why smoking among adolescent girls is an important issue in Ghana.

- In the background part of the abstract, the authors mentioned “…, and in particular narrowing gender gaps, highlight the need for interventions to prevent and/or reduce tobacco use among adolescent girls”. It is not clear what narrowing gender gaps mean here. Are we saying boys have higher smoking prevalence and girls are catching up? If this is the case, then in the Introduction, the authors need to expand on this part with proper citations.
- In the last paragraph of the Introduction, the authors mentioned “only 1.6% of girls reported ever tried smoking at baseline and 0.1% having smoked in the past 30 days.” These rates are extremely low, especially compared to those in developing countries. For example, past 30-day cigarette smoking among middles school students in the US was 1.1% in 2023. ^1^ Why is smoking among adolescent girls in Ghana an important topic to study? Has the smoking prevalence increased in the recent years? The authors need to add more background information and justification.

2. While the machine learning techniques are cool and novel, it is not clear how the findings advance our existing knowledge. Many of the factors identified, such as peer influence (is smoking common among friends) and exposure to smoking (most activities involve smoking cigarettes), are well-known factors that are associated with smoking initiation. The authors need to compare their results with existing literature and highlight their contribution. This can be done by adding a paragraph or two in the Discussion.

 3. To demonstrate the advantage or benefits of machine learning techniques, the authors could conduct the same analysis using convention regression approaches and compare the results. For example, logistic regressions can also identify risk factors that are significantly associated with ever smoking or past 30-day smoking.

Is the work clearly and accurately presented and does it cite the current literature?

Partly

If applicable, is the statistical analysis and its interpretation appropriate?

Yes

Are all the source data underlying the results available to ensure full reproducibility?

Yes

Is the study design appropriate and is the work technically sound?

Yes

Are the conclusions drawn adequately supported by the results?

Yes

Are sufficient details of methods and analysis provided to allow replication by others?

Yes

Reviewer Expertise:

Tobacco control; policy evaluation; health economics; modeling and simulation.

I confirm that I have read this submission and believe that I have an appropriate level of expertise to confirm that it is of an acceptable scientific standard, however I have significant reservations, as outlined above.

## References

[1] : Tobacco Product Use Among U.S. Middle and High School Students - National Youth Tobacco Survey, 2023. *MMWR Morb Mortal Wkly Rep* .2023;72(44) : 10.15585/mmwr.mm7244a1 1173-1182 10.15585/mmwr.mm7244a1 37917558 PMC10629751

# Response

**Published:** 2024-11-19

**Authors:** Sara Flanagan

**Affiliations:** ideas42, USA

1. There is a lack of information or justification of why smoking among adolescent girls is an important issue in Ghana.

- In the background part of the abstract, the authors mentioned “…, and in particular narrowing gender gaps, highlight the need for interventions to prevent and/or reduce tobacco use among adolescent girls”. It is not clear what narrowing gender gaps mean here. Are we saying boys have higher smoking prevalence and girls are catching up? If this is the case, then in the Introduction, the authors need to expand on this part with proper citations.
  - **Response**: We have expanded on this background information in the Introduction section and added citations.
- In the last paragraph of the Introduction, the authors mentioned “only 1.6% of girls reported ever tried smoking at baseline and 0.1% having smoked in the past 30 days.” These rates are extremely low, especially compared to those in developing countries. For example, past 30-day cigarette smoking among middles school students in the US was 1.1% in 2023. ^1^ Why is smoking among adolescent girls in Ghana an important topic to study? Has the smoking prevalence increased in the recent years? The authors need to add more background information and justification.
  - **Response**: Other studies had suggested increasing tobacco use, and in particular shisha, among adolescent girls in Ghana. We have expanded on this background information in the Introduction section and added citations.

2. While the machine learning techniques are cool and novel, it is not clear how the findings advance our existing knowledge. Many of the factors identified, such as peer influence (is smoking common among friends) and exposure to smoking (most activities involve smoking cigarettes), are well-known factors that are associated with smoking initiation. The authors need to compare their results with existing literature and highlight their contribution. This can be done by adding a paragraph or two in the Discussion.

- **Response**: We recognize the word limit for research notes is a constraint to significantly expanding the Discussion, but we have edited the text to specify that these insights have programmatic value for planning interventions that are responsive to this specific population and context. We also encourage readers to refer to our recently published qualitative research study (cited) for a more extensive discussion of these findings relative to the existing tobacco literature.

3. To demonstrate the advantage or benefits of machine learning techniques, the authors could conduct the same analysis using convention regression approaches and compare the results. For example, logistic regressions can also identify risk factors that are significantly associated with ever smoking or past 30-day smoking.

- **Response**: Comparing our model against a logistic regression model may not be the best comparison, since Random Forests can better leverage the additional samples created through SMOTE as it builds multiple trees on different subsets of the data, which may help to generalize better. Random Forests also provide additional benefits over logistic regression. Random Forests provide insights into the importance of different predictors, allowing you to identify which features contribute most to the prediction, whereas logistic regression provides coefficients that can be harder to interpret, especially with interactions. Random Forests are also less prone to overfitting, especially in complex datasets, because they aggregate predictions from multiple trees, which helps to smooth out noise.
